# Giant Neurofibroma of the Left Median Nerve Associated With Damage of the Ipsilateral Distal Radius

**DOI:** 10.7759/cureus.20294

**Published:** 2021-12-09

**Authors:** Ioannis E Kougioumtzis, Antonia Barmpitsioti, Stylianos Tottas, Alexandra Giatromanolaki, Georgios I Drosos

**Affiliations:** 1 Academic Orthopaedic Department, Democritus University of Thrace, University General Hospital of Alexandroupolis, Alexandroupoli, GRC; 2 Department of Orthopaedics, KAT Attica General Hospital, Kifisia, GRC; 3 Academic Pathology Department, Democritus University of Thrace, University General Hospital of Alexandroupolis, Alexandroupoli, GRC

**Keywords:** bone damage, distal radius, median nerve, neurofibroma, neural sheath tumor

## Abstract

A 74-year-old Caucasian woman presented with a large mass on her left distal radius, which had previously caused a fracture of the bone at this site and the palmar site that was treated with external fixation a year ago. The patient did not mention tumor-related family history and other neoplasms before the fracture of the distal radius. She noticed that the gradually growing mass had appeared after the fracture treatment. A thorough evaluation of the lesion confirmed the diagnosis of a large benign neurofibroma with distal radius impairment. The diagnostic and therapeutic procedure included the complete excision of the tumor and a six-week cast immobilization of the radius. On the final follow-up two years postoperatively, her clinical situation was satisfactory with no signs of recurrence. Although rare, isolated benign neurofibromas of enormous sizes are associated with bone damage. In our view, immediate surgical excision is crucial and enables total recovery postoperatively.

## Introduction

Neurofibromas (NFs) are defined as benign peripheral nerve sheath tumors (PNSTs). PNSTs comprise about 5% and 2% of all tumors of the upper extremity in adults and children, respectively [1-3]. The most common benign PNSTs include neurofibroma and schwannoma, accounting for approximately 10% to 12% of all benign soft tissue neoplasms. NFs manifest in patients of all ages. According to the literature, they may occur in the upper and lower extremities as well [2,4]. The predominant gender is female and the mean age of patients varies [2,3]. Previous studies have shown an association between NF and neurofibromatosis type 1 (NF-1). However, the majority of these cases are not associated with NF-1 (ranging from 60% to 90%) [2,5].

Further, bony involvement is extremely rare, especially in the upper extremities [6,7]. Patients with NFs pose a diagnostic and therapeutic challenge. According to the literature, their macroscopic and microscopic appearance can vary broadly [7,8]. Although the diagnosis is established with imaging in the majority of the cases, the gold standard for diagnosis remains histopathological examination [5].

Here, we report an uncommon case of a huge NF of the left median nerve associated with the destruction of the ipsilateral distal radius. In addition, we describe its clinical manifestation, the diagnostic workup, and the appropriate treatment.

## Case presentation

**Figure 1:**
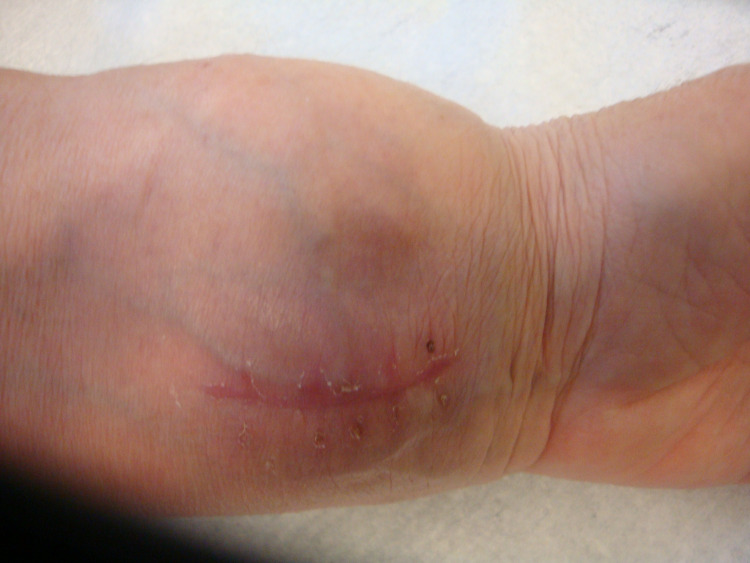
The scar of the incision due to the prior biopsy and a palpable mass on the flexor surface of the left wrist.

**Figure 2:**
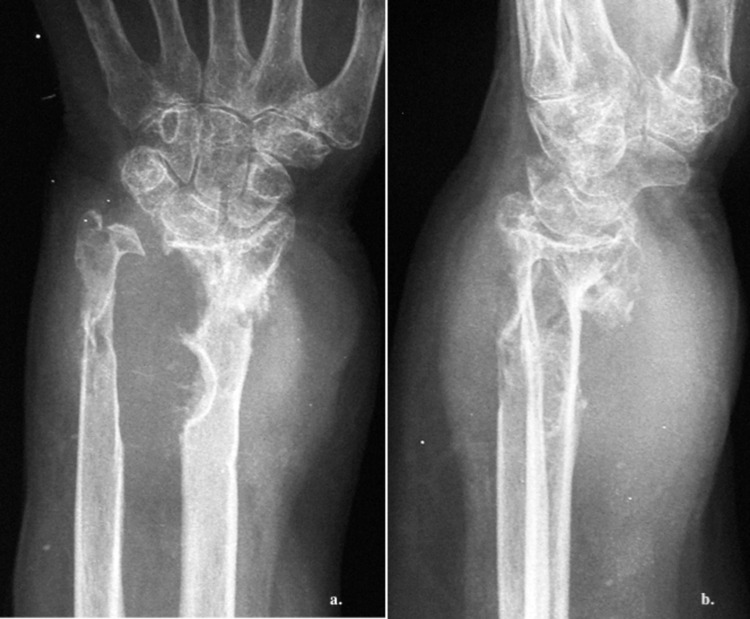
Radiological imaging of the left wrist showing a large soft tissue lesion causing destruction of the ipsilateral distal radius. (a) Anteroposterior and (b) mediolateral images.

A 74-year-old Caucasian woman presented to our hospital with a mass in the left wrist (Figure 1). Regarding her history of trauma, she fell on her outstretched hand one year ago and sustained a left-sided distal radius fracture. The fracture was reduced and stabilized through external fixation in another hospital. However, postoperatively, the patient noticed a gradually increasing palpable mass at the volar side of the same wrist. Although she had never experienced her hand symptoms prior to the fracture, postoperatively, she complained of occasionally occurring nocturnal pain. Regarding her family history, there were no reports of related systematic or neoplasm diseases. On clinical examination, percussion over the mass produced a Tinel-like sensation along the median nerve. Neither motor weakness nor muscular atrophy was observed. On radiographic imaging, the characteristic pattern of a large soft tissue mass with distal radius damage was noted (Figure 2). Other laboratory findings were normal.

**Figure 3:**
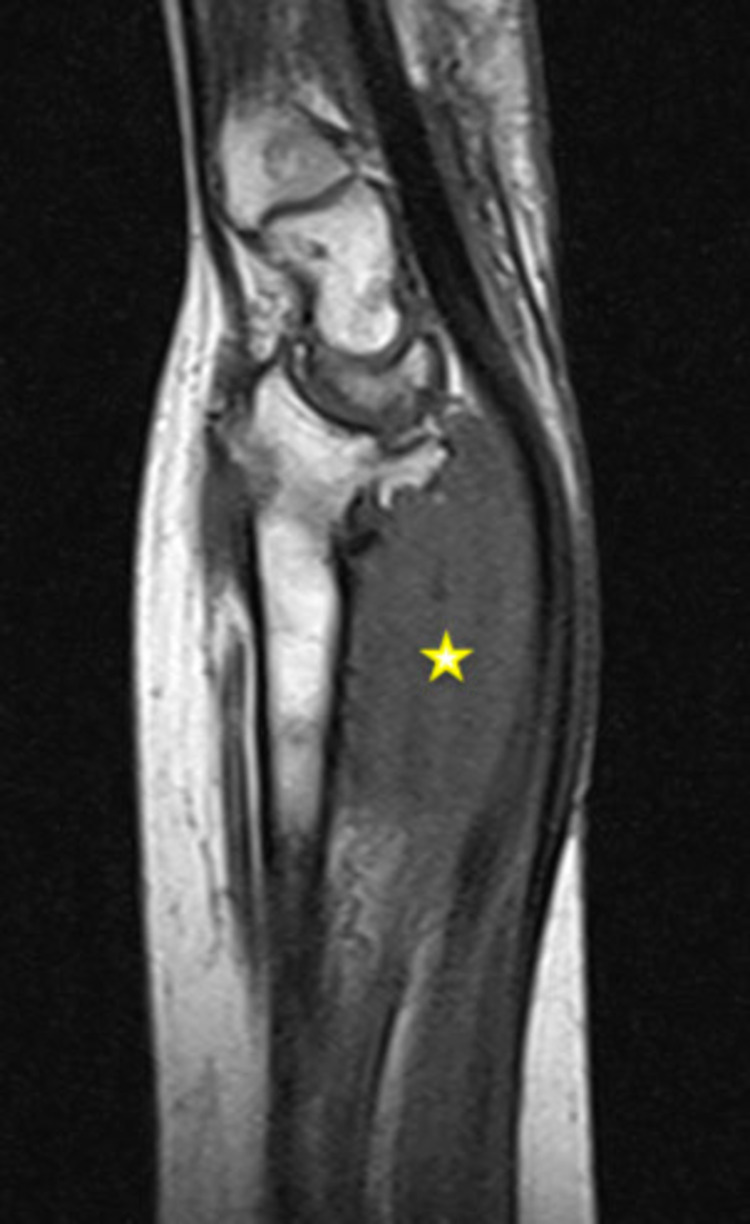
Coronal view of the left wrist (MRI; T1W image). The lesion (yellow asterisk) shows a homogenous low signal, slightly lower to the adjacent muscle. MRI: magnetic resonance imaging; T1W: T1-weighted

**Figure 4:**
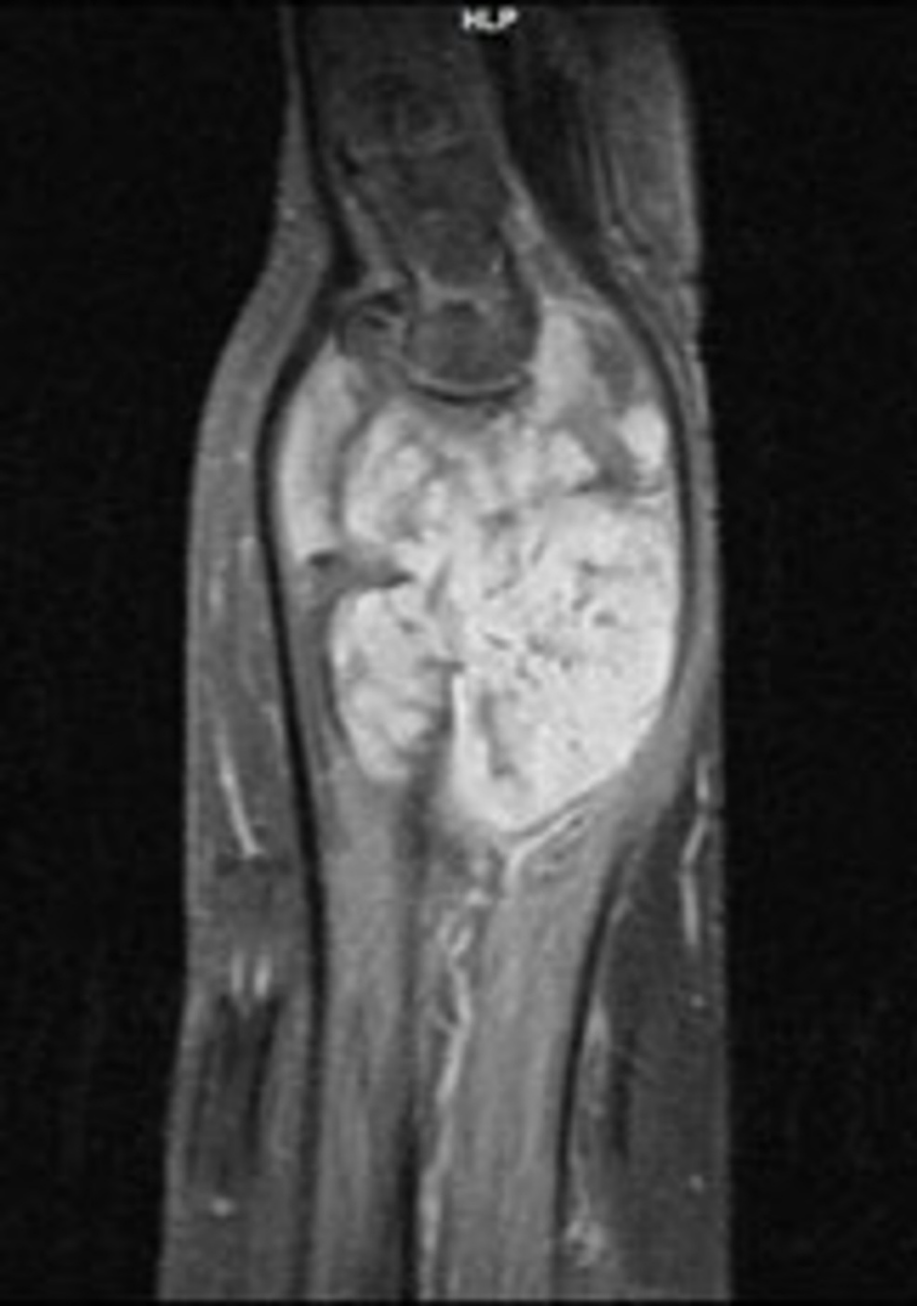
Coronal view of the left wrist (MRI; T2W image). The lesion shows the characteristic “target pattern.” MRI: magnetic resonance imaging; T2W: T2-weighted

**Figure 5:**
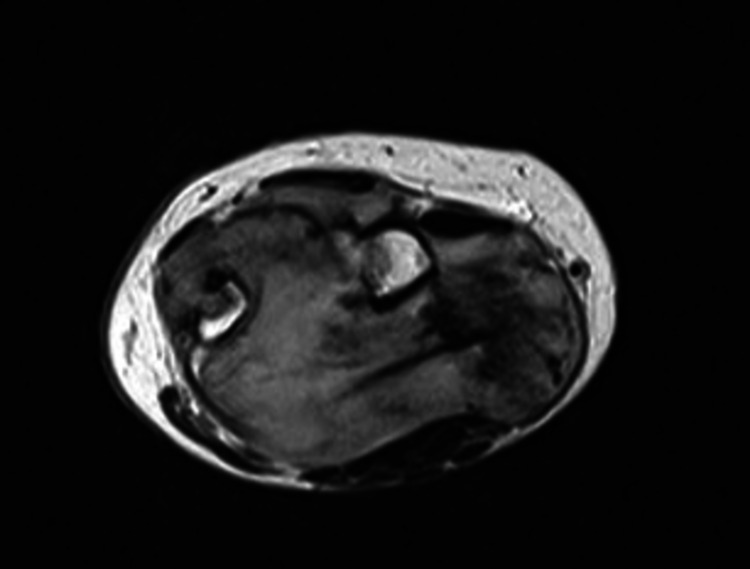
Axial view of the left wrist (MRI; T1W image). The mass shows an increased and heterogenous signal density. MRI: magnetic resonance imaging; T1W: T1-weighted

**Figure 6:**
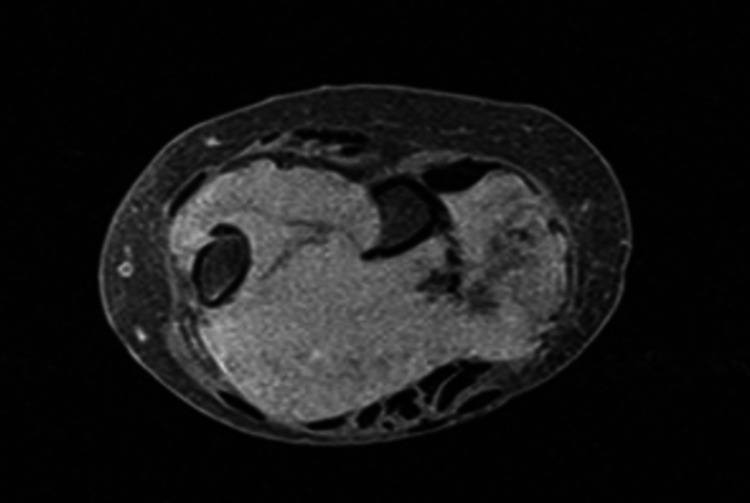
Axial view of the left wrist (MRI; T2W image). The lesion is round and well-circumscribed with an apparent enhancement of the periphery. MRI: magnetic resonance imaging; T2W: T2-weighted

The magnetic resonance imaging (MRI) revealed a 4.5 cm × 6.5 cm × 6.5 cm, well-circumscribed mass with bone damage in both the ulna and the radius. The lesion showed a relatively homogeneous low signal, slightly lower compared to that of the adjacent muscles, on T1-weighted (T1W) images. No edema around the tumor was present, instead, there was a zone of adipose tissue surrounding the tumor (Figures 3, 5). On T2-weighted (T2W) images, the lesion had a relatively homogenous high signal, which was slightly higher than that of the surrounding muscles (Figures 4, 6). The characteristic “target pattern” was observed, revealing a hyperintense rim surrounding a central area of the reduced signal.

**Figure 7:**
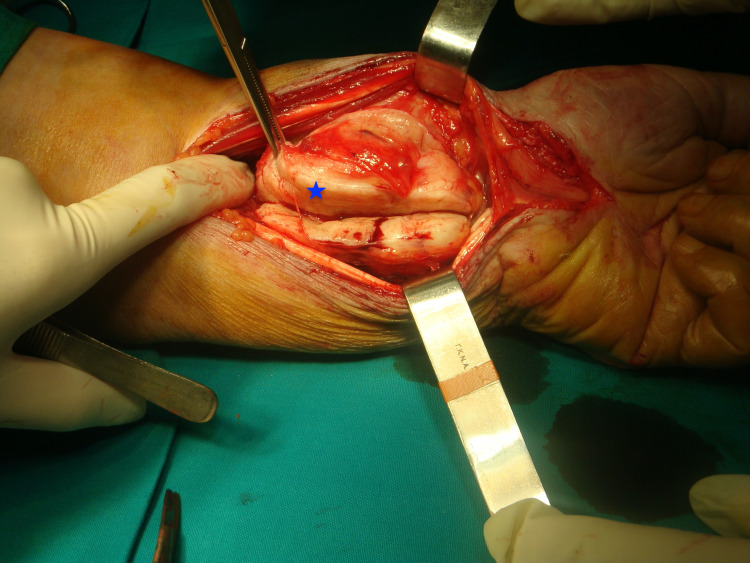
Surgical incision over the enlarged left wrist reveals a well-formed, encapsulated mass, with an eccentric position along the median nerve (blue asterisk).

**Figure 8:**
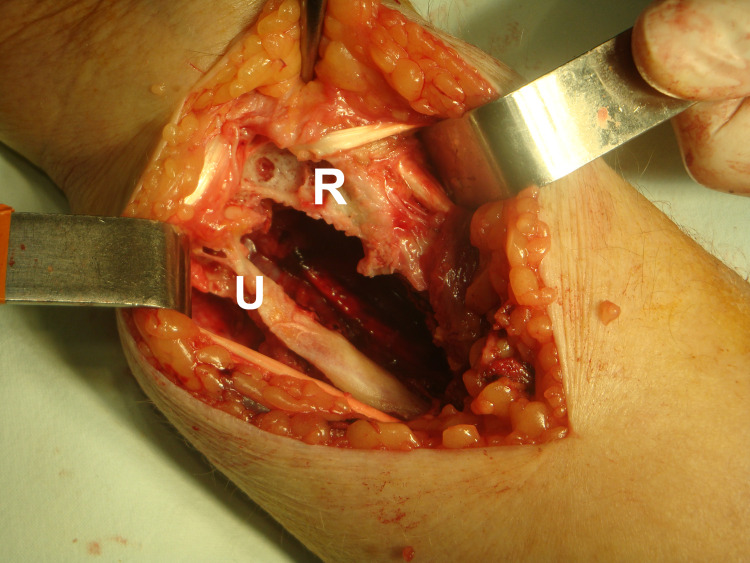
After complete removal of the mass, the extent of the distal ulna and radius damage is clear. R: radius; U: ulna

**Figure 9:**
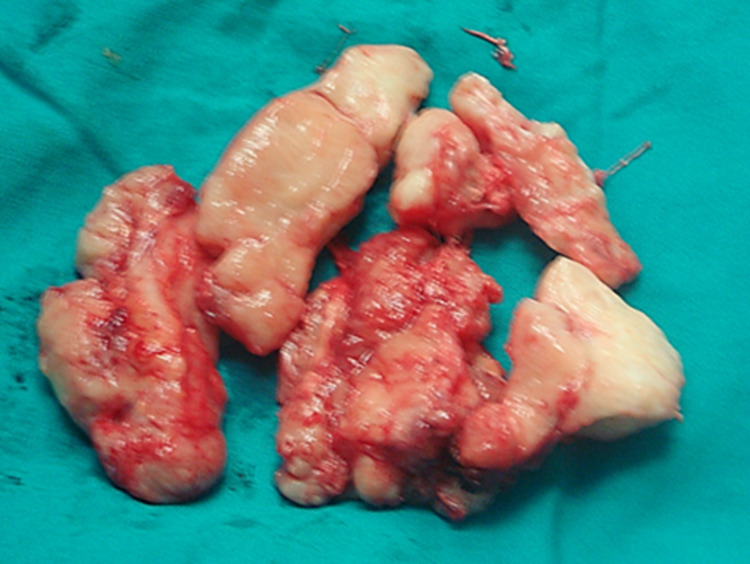
Surgical specimen of the resected tumor with a gray color and a firm consistency.

We performed a biopsy before the complete surgical excision. For the final excision, a longitudinal incision centered over the tumor bulk was made, without releasing the transverse ligament of the carpal tunnel. The lesion was revealed in an eccentric position along the median nerve. A marginal tumor excision with preservation of the median nerve was achieved after careful surgical manipulations. After the complete excision of the mass, clear damage to the distal ulna and radius was seen (Figures 7-9). The defect of the bone and soft tissue was postoperatively protected with a forearm splint for six weeks.

**Figure 10:**
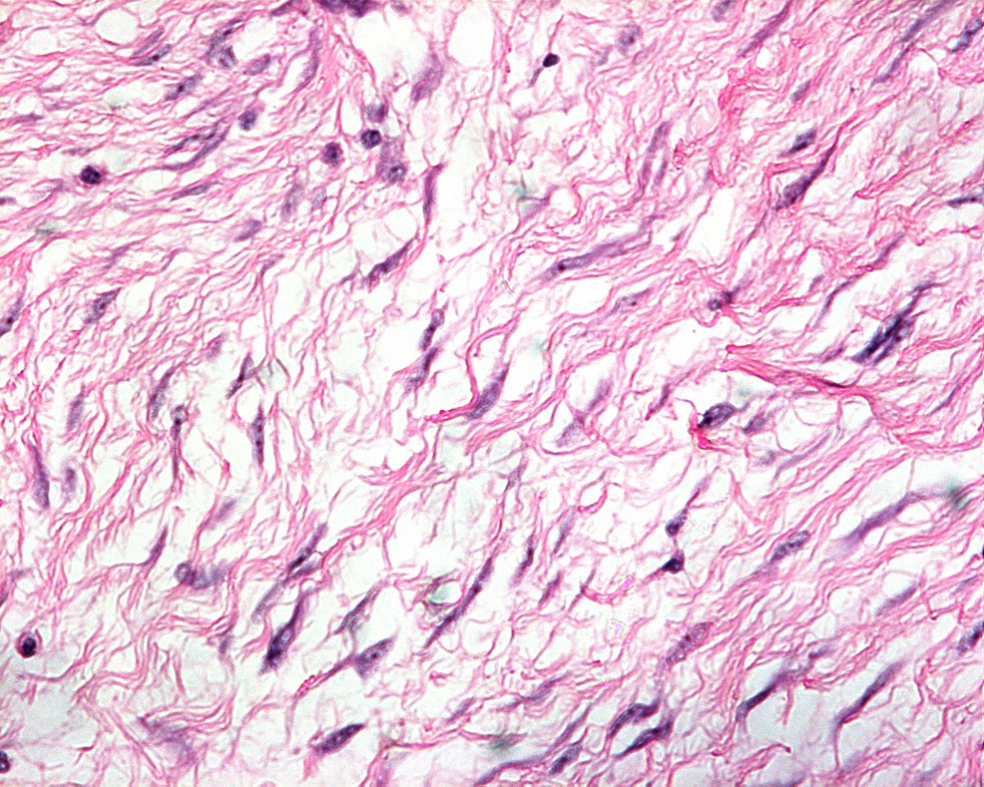
Tumor cells with curved and elongated nuclei admixed with fibroblasts in a collagen-rich matrix (H&E; ×100). H&E: hematoxylin and eosin

The resected tumor samples (bone and soft tissue) were sent to the pathology department for evaluation and diagnosis. Macroscopically, the specimens were gray in appearance with solid consistency and measured 2.5 × 7 cm with co-resected bones and soft tissues. Histologically, the soft tissue tumor cells were composed of curved and elongated nuclei admixed with fibroblasts in a collagen-rich matrix. The bone did not reveal pathology related to soft tissue tumor cells or other pathology. The final pathology report was consistent with a solitary localized benign neurofibroma (Figure 10).

**Figure 11:**
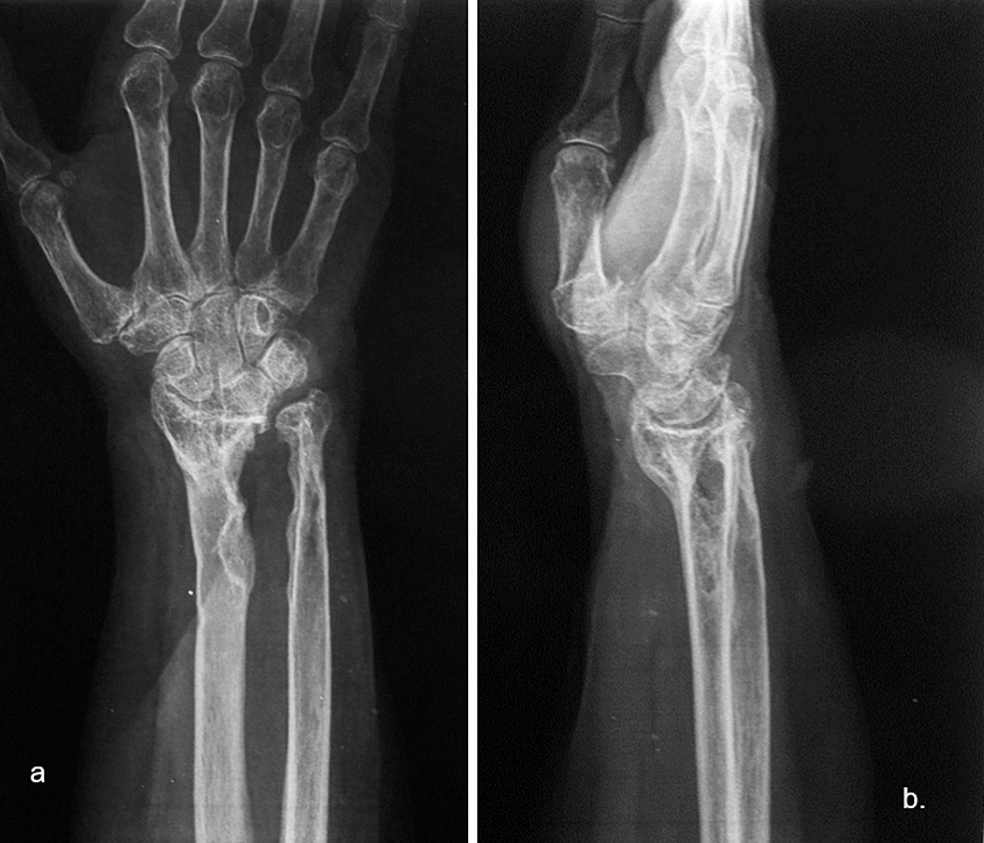
Postoperative X-rays images one year postoperatively. (a) Anteroposterior and (b) mediolateral.

Postoperatively, the patient underwent physiotherapy. On the follow-up X-ray imaging at one year postoperatively (Figure 11), her clinical situation was satisfactory, with no pain upon palpation and sufficient motion at one and two-year follow-up.

## Discussion

The precise pathogenetic mechanism underlying the development of NFs in the median nerve has not been fully elucidated [7,9]. Therefore, we investigated whether the natural history of sudden or gradual onset in NFs can be associated with a previous traumatic event [6,7].

Histological examination remains a crucial step toward the final diagnosis [3,7]. The recent World Health Organization (WHO) Classification of Soft Tissue and Bone tumors highlighted that this lesion is accompanied by intraneural or extraneural findings. Similar to our case, solitary lesions, which comprise approximately 90% of all NFs, appear nodular, polypoid, and circumscribed [3]. Kubiena et al. [1] showed that these lesions arise from the cells of the nerve sheath and engulf some nerve fascicles. However, bone destruction due to a benign NF is unusual. This characteristic presentation of bone damage is commonly seen in schwannomas, neuromas, and malignant tumors [10].

The size and the infiltration of the surrounding structures determine the precise clinical symptoms [11]. The sign of a solitary lesion arising in or next to a nerve steadily increases pain. However, symptoms of nocturnal or spontaneous pain are usually attenuated in malignant tumors. In addition, deep lesions usually present with motor power weakness, sensory deficit or paresthesia, and severe pain [1,7]. Several studies have underlined the difficulty in diagnosing a tumor of neural sheath origin and concluded that such a diagnosis remains a challenge. In the differential diagnosis using MRI findings, the following tumors can be considered: begin solitary neoplasms such as lipomas, fibromas, xanthomas, ganglion tumors, mucous cysts, glomus tumors, giant cell tumors of the tendon sheath, vascular tumors, as well as post-traumatic neuromas, in addition to low-grade malignant neoplasms [7]. Nevertheless, the final diagnosis is confirmed on histological examination. On the other hand, symptomatic neurofibromatosis is directly linked with multiple lesions which assists in the final diagnosis [3,7].

Imaging studies are valuable for preoperative assessment. Plain radiographs are an initial step toward the diagnosis. This was also seen in our case as the patient had extended bone damage, which was identified on the radiologic examination. In general, ultrasonography (US) is a useful diagnostic method to differentiate between a benign and a malignant lesion [5]. However, due to the low accuracy of US, current studies employ a combination of US and MRI, which provides accurate information for both intraneural and extraneural lesions [12]. Currently, MRI is the gold standard for preoperative assessment [7,11]. On T1W images, NFs show a low-to-intermediate signal, and on T2W images they show a high signal. These findings were also evident in our patient. As described in the majority of previous studies, there is a characteristic pattern for NF, the “target sign,” which refers to a homogenous hyperintense region [5,4,13].

A challenging step in the treatment is the complete tumor excision and the simultaneous preservation of nerve function [2]. Regarding the tumor excision, we utilized microsurgical excision techniques [5], including meticulous dissection of the nerve fascicles, resulting in minimal damage; and preservation of function and anatomical continuity of the nerve. On the other hand, wide local dissection is difficult in patients presenting with large masses and infiltration of the surrounding soft tissue and bone [2].

Our patient had no complications at the two-year follow-up. Postoperative recovery and good prognosis in such cases depend on surgical skills and lesion patterns [2].

## Conclusions

The assessment and diagnosis of NFs remain challenging. The available evidence indicates that imaging features are a debatable topic regarding the diagnostic accuracy of nerve tumors. NFs are soft tissue lesions that cause bone damage on rare occasions. However, clinicians should consider them as a possible cause of bone damage. Finally, we believe that complete surgical excision of such tumors can provide a satisfactory outcome.
